# Genome Sequences of Porcine Epidemic Diarrhea Virus: *In Vivo* and *In Vitro* Phenotypes

**DOI:** 10.1128/genomeA.00503-14

**Published:** 2014-06-12

**Authors:** Paulraj K. Lawrence, Eric Bumgardner, Russell F. Bey, Douglas Stine, Roger E. Bumgarner

**Affiliations:** a Newport Laboratories Inc., Worthington, Minnesota, USA; b Department of Microbiology, University of Washington, Seattle, Washington, USA

## Abstract

Since the outbreak of porcine epidemic diarrhea virus (PEDV) in May 2013, U.S. swine producers have lost almost five million baby pigs. In an attempt to understand the evolution of PEDV in the United States and possibly develop a control strategy, we compared the genome sequences of a PEDV strain isolated from an infected piglet against its *in vitro* adapted version. The original PEDV strain was grown in Vero cells and passed 10 times serially in a MARC145 cell line. The sequence analysis of the native PEDV strain and *in vitro* passaged virus shows that the cell culture adaptation specifically modifies PEDV spike protein whereas the open reading frame 1a/b (ORF1a/b)-encoded polyprotein, the nucleoprotein, NS3B (ORF3), and membrane and envelope proteins remain unchanged.

## GENOME ANNOUNCEMENT

Porcine epidemic diarrhea virus (PEDV) causes a severe and highly contagious swine disease. While older pigs have a chance of survival, 80 to 100 percent of PEDV-infected piglets die within 24 h of being infected. PEDV spreads primarily through fecal-oral contact (1, 2). Once the virus is internalized, it destroys the lining of piglets’ intestines, making them incapable of digesting and deriving nutrition from milk and feed (1). The virus causes diarrhea, vomiting, and death from dehydration and starvation (2).

PEDV is a member of the *Coronavirinae* subfamily and belongs to the *Alphacoronavirus* genus. Its genomic size ranges from approximately 26 to 32 kb, which is relatively large for an RNA virus. Although vaccines for PEDV exist in China, Japan, and South Korea, there is no approved vaccine in the United States or Europe (3). Furthermore, PEDV is still evolving within the U.S. swine population.

This report briefly describes the comparison of genome sequences of a PEDV strain isolated from small intestine samples of an infected piglet and its *in vitro* adapted version. The original PEDV strain was dubbed NPL-PEDV/2013, grown in Vero cells, and passed 10 times in a MARC145 cell line. The serial *in vitro* passage strain was named NPL-PEDV/2013/P10. The total viral RNA was extracted by TRIzol LS reagent and sequenced by Sanger dideoxy sequencing using a primer walking technique. The raw sequences were imported into the Geneious assembler (Biomatters, CA), assembled, annotated, and compared against each other using USA/Colorado/2013 (GenBank accession no. KF272920) as a reference sequence.

The whole-genome sequences of NPL-PEDV/2013 and NPL-PEDV/2013/P10 contain 28,038 and 28,025 nucleotides (nt), respectively, including the 5′ and 3′ untranslated regions (UTR). The NPL-PEDV/2013 genome shares 99% identity with all the U.S. isolates sequenced to date and many Chinese isolates as well. The top three BLAST hits were against U.S. isolates, USA/Colorado/2013 (GenBank accession no. KF272920), IA1 (GenBank accession no. KF468753.1), and an isolate from Iowa, 13-019349 (GenBank accession no. KF267450.1). The NPL-PEDV/2013 isolate also shares 99% identity with the Chinese outbreak isolate AH2012 (GenBank accession no. KC210145).

When the NPL-PEDV/2013/P10 strain was compared against NPL-PEDV/2013 , the open reading frame 1a/b (ORF1a/b) polyprotein, the nucleoprotein, NS3B, and membrane and envelope proteins were found to be 100% identical at the amino acid level. In contrast, the spike gene contains six nonsynonymous single nucleotide polymorphisms, resulting in amino acid (aa) substitutions in the following positions: 375 (F→L), 486 (T→P), 856 (D→E), 1081 (A→V), 1099 (A→S), and 1253 (Y→D). The S1 domain of spike protein contains 2 aa substitutions, whereas the S2 domain contains 4 aa substitutions. PEDV has been shown to use porcine aminopeptidase N (pAPN) as the major receptor for cell entry (4, 5). However, Vero and MARC145 cells lack pAPN, clearly indicating that other receptors or receptor-independent pathways may be used for entry (6). The spike protein in its trimeric conformation interacts with the cell receptor and contains numerous neutralizing antibody binding epitopes (7). Analysis of the spike by PeptideCutter (http://web.expasy.org/peptide_cutter/) shows that the native spike protein of NPL-PEDV/2013 has 63 trypsin and 2 chymotrypsin cleavage sites at 100% efficiency whereas NPL-PEDV/2013/P10 has lost one trypsin cleavage site but the number of chymotrypsin sites remain unchanged. This indicates that cell culture adaptation specifically modifies the PEDV spike protein; however, the immunological implications are unknown.

### Nucleotide sequence accession numbers.

The whole-genome sequences of the NPL-PEDV/2013 and NPL-PEDV/2013/P10 strains have been deposited at DDBJ/EMBL/GenBank under accession no. KJ778615 and KJ778616.

## References

[1] Pospischil A Stuedli A Kiupel M 2002 Diagnostic notes update on porcine epidemic diarrhea. J. Swine Health Prod. 10:81–85

[2] Song D Park B 2012 Porcine epidemic diarrhoea virus: a comprehensive review of molecular epidemiology, diagnosis, and vaccines. Virus Genes 44:167–175. 10.1007/s11262-012-0713-1 22270324 PMC7089188

[3] U.S. Department of Agriculture 2013 Technical note: porcine epidemic diarrhea (PED). U.S. Department of Agriculture, Fort Collins, CO http://www.aphis.usda.gov/animal_health/animal_dis_spec/swine/downloads/ped_tech_note.pdf

[4] Nam E Lee C 2010 Contribution of the porcine aminopeptidase N (CD13) receptor density to porcine epidemic diarrhea virus infection. Vet. Microbiol. 144:41–50. 10.1016/j.vetmic.2009.12.024 20074871 PMC7117352

[5] Li BX Ge JW Li YJ 2007 Porcine aminopeptidase N is a functional receptor for the PEDV coronavirus. Virology 365:166–172. 10.1016/j.virol.2007.03.031 17467767 PMC7103304

[6] Taguchi F Matsuyama S 2002 Soluble receptor potentiates receptor-independent infection by murine coronavirus. J. Virol. 76:950–958. 10.1128/JVI.76.3.950-958.2002 11773370 PMC135807

[7] Belouzard S Millet JK Licitra BN Whittaker GR 2012 Mechanisms of coronavirus cell entry mediated by the viral spike protein. Viruses 4:1011–1033 22816037 10.3390/v4061011 PMC3397359

